# Pediatric Hemophilic Arthropathy of the Knee: Treatment With Circular External Fixator and Intra-articular Injection of Platelet-Rich Plasma

**DOI:** 10.7759/cureus.56198

**Published:** 2024-03-14

**Authors:** Eleni Pappa, Maria Giannakopoulou, Aikaterini G Michalopoulou, Anastasia Pilichou, Athina Dettoraki, Dimitrios Syrengelas, Marios Antoniadis, Helen Pergantou, John Anastasopoulos

**Affiliations:** 1 Orthopaedics, 5th Orthopaedic Department, KAT General Hospital, Athens, GRC; 2 Orthopaedics, 2nd Orthopaedic Department, Agia Sofia Children's Hospital, Athens, GRC; 3 Haemophilia Centre/Haemostasis and Thrombosis Unit, Agia Sofia Children’s Hospital, Athens, GRC; 4 Haemophilia Centre/Haemostasis and Thrombosis Unit, Agia Sofia Children's Hospital, Athens, GRC

**Keywords:** prp injection, flexion contracture, ilizarov fixator, knee, hemophilia

## Abstract

There are limited reports about managing knee flexion contracture (KFC) due to hemophilic hemarthrosis with the Ilizarov technique and platelet-rich plasma intraarticular injection administration. This article aims to describe a case of KFC treated with a circular external fixator and intraarticular administration of platelet-rich plasma in a pediatric patient.

A 12-year-old male patient suffering from hemophilia A was being monitored by our department due to knee effusions. Extensive knee flexion contracture of the left knee was seen. The Ilizarov technique was chosen for surgical management of the worsening knee flexion contracture. The duration of distraction was six weeks. Due to localized pain and functional impairment, intra-articular administration of platelet-rich plasma (PRP) was applied twice, on the first month after the circular frame removal and at a six-month follow-up, with clinical and functional improvement.

Our clinical case report demonstrates that PRP intra-articular injections are likely to provide an improvement in pain and knee joint function, as well as joint hyperemia, even in the case of already established knee flexion contracture, which was managed with a circular distraction device. However, more studies regarding the Ilizarov technique and the PRP intraarticular administration are needed for a protocol to be established for the management of the hemophilic knee joint in the pediatric population.

## Introduction

Hemophilia is an X-linked coagulation disorder because of a congenital deficiency of factor VIII, leading to hemophilia A, or IX, which leads to hemophilia B. Hemarthroses constitute approximately 85% of all bleeding episodes in patients with hemophilia (PwH) and most commonly involve the ankles, knees, and elbows. Intravenous administration of prophylactic treatment with factor VIII or IX concentrates has significantly reduced the incidence of joint bleeds with an improvement in the quality of life for patients with hemophilia (PwH). Early findings of arthropathy include effusion, synovial hypertrophy followed by osteochondral changes, bone erosions, and subchondral cyst formation, as well as the presence of flexure contracture of the joints in later stages such as the knee joint [1].

The Ilizarov technique is a method of skeletal traction that steadily distracts the joint, reducing the possibility of any neurovascular or skin injury. For that reason, it is a popular alternative to open surgery and arthrotomy in such cases of hemophilic arthropathy in adults. However, in the current literature, there have been a few studies reporting the management of KFC with circular external fixators in the pediatric population [2].

Regarding intra‐articular therapy, hyaluronic acid (HA) has been used in clinical practice for the treatment of hemophilic arthropathy. Fernandez‐Palazzi et al. first used intra‐articular HA injections for patients with hemophilia with arthropathy signing improving functional results [3]. Furthermore, intra‐articular platelet‐rich plasma (PRP) injection includes a natural concentrate of autologous growth factors from the blood, which are popular for their anti-inflammatory potential. Intra‐articular PRP injection therapy has been extensively applied in the management of osteoarthritis but regarding the treatment for hemophilic arthropathy, it is rarely reported in current studies. No serious adverse effects of PRP have been mentioned, however, the use of multiple PRP injections in osteoarthritic patients may increase the risk of infection and joint swelling as proposed by many case series. Moreover, there is evidence showing that HA and PRP have different mechanisms in the treatment of arthropathy. Laboratory findings relevant to comparing HA and PRP treatment suggested that HA and PRP decrease inflammation in a different pathway, as demonstrated in the different expressions of cytokines and matrix metalloproteinases. Consequently, the beneficial effect of PRP in the treatment of hemophilic arthropathy suggests a potential alternative therapy that may be useful to patients who were unresponsive to HA treatment in the past [4].

This case presentation aims to shed light on the management of knee flexion contracture due to hemophilic arthropathy in a pediatric patient, treated with both circular external fixator and intra-articular administration of PRP.

This article was previously presented as a meeting abstract at the WFH Comprehensive Care Summit on May 10-12 2023.

## Case presentation

**Figure 1:**
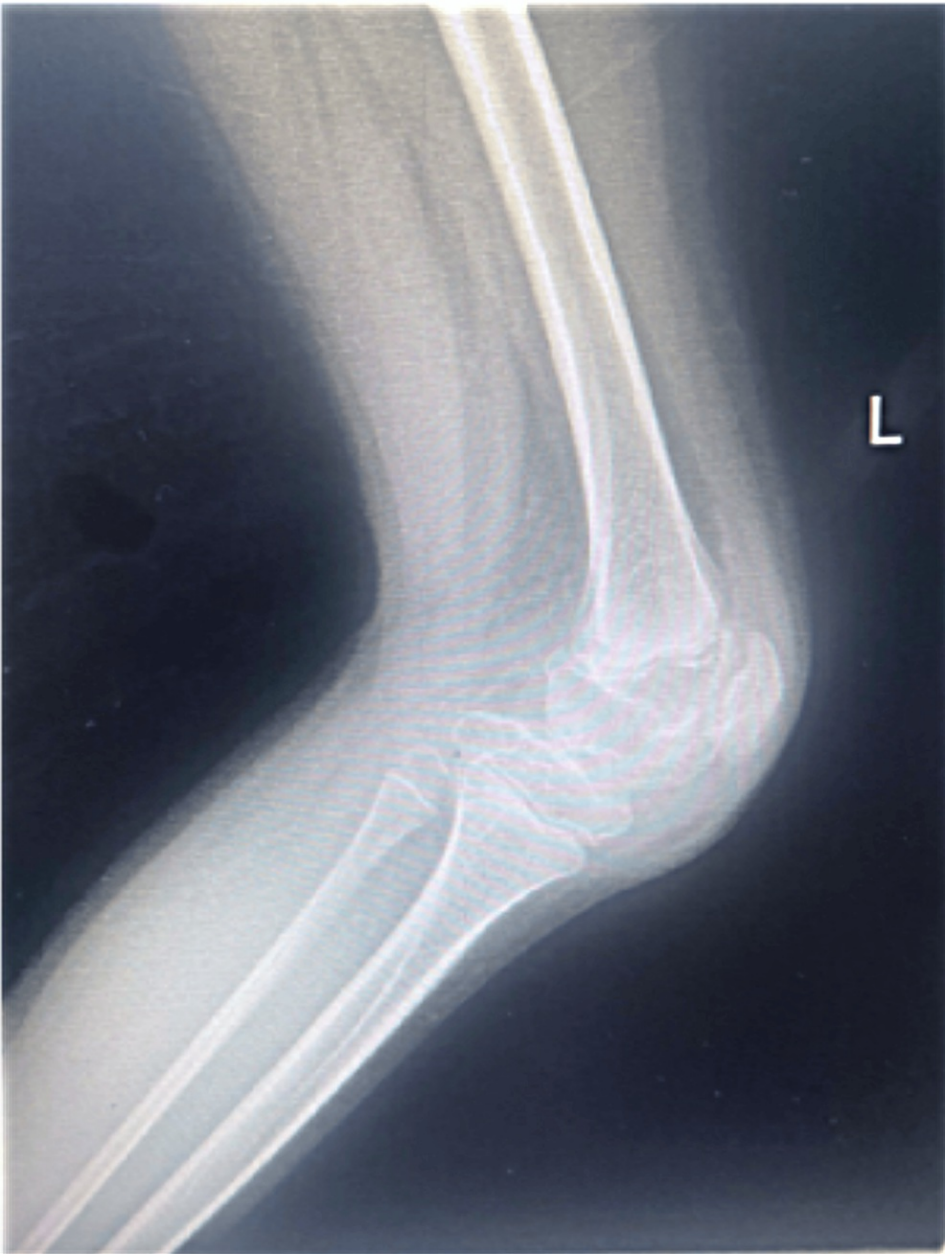
Preoperative lateral X-ray of the left hemophilic knee

**Figure 2:**
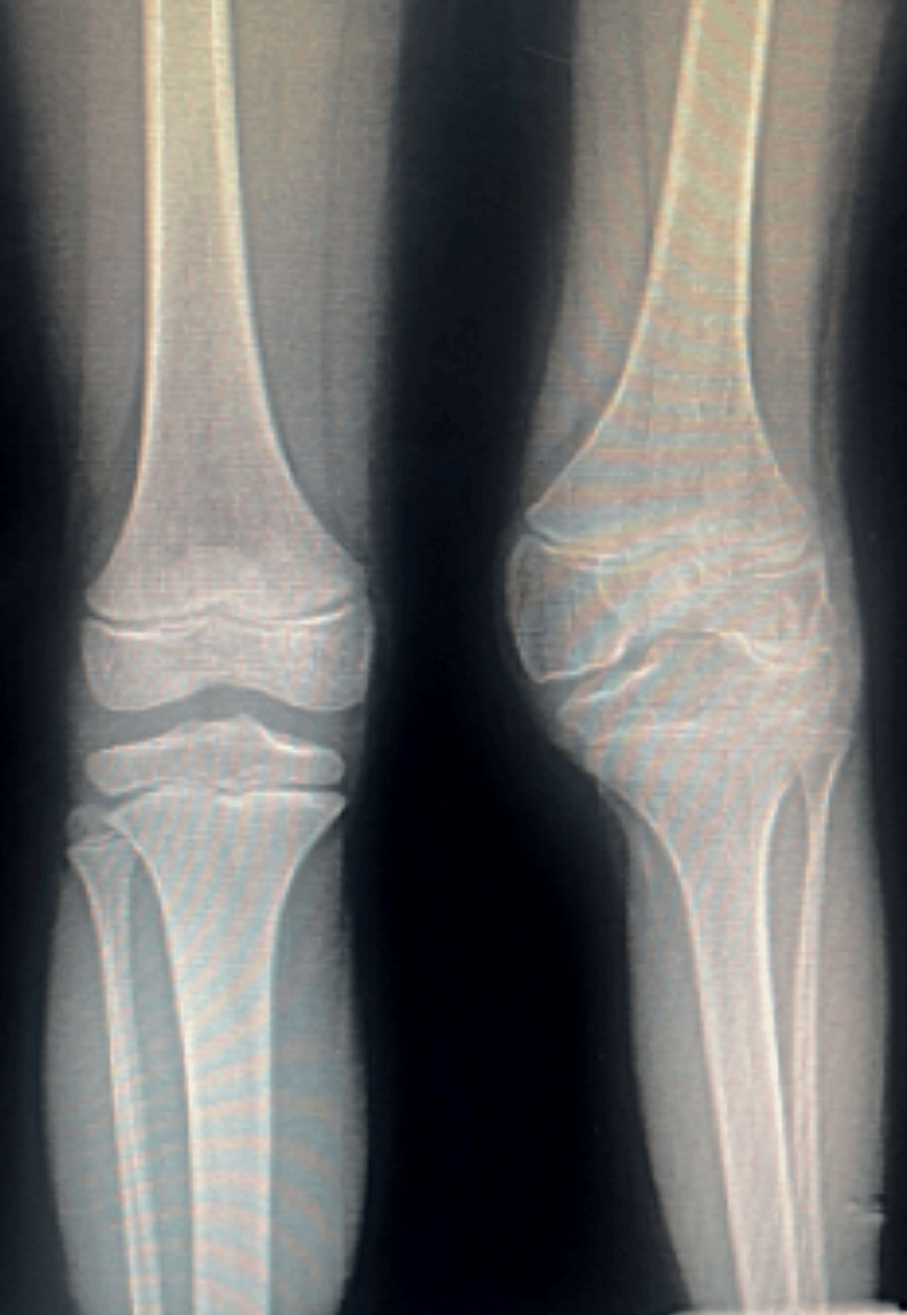
Preoperative anterior X-ray of the hemophilic knee

**Figure 3:**
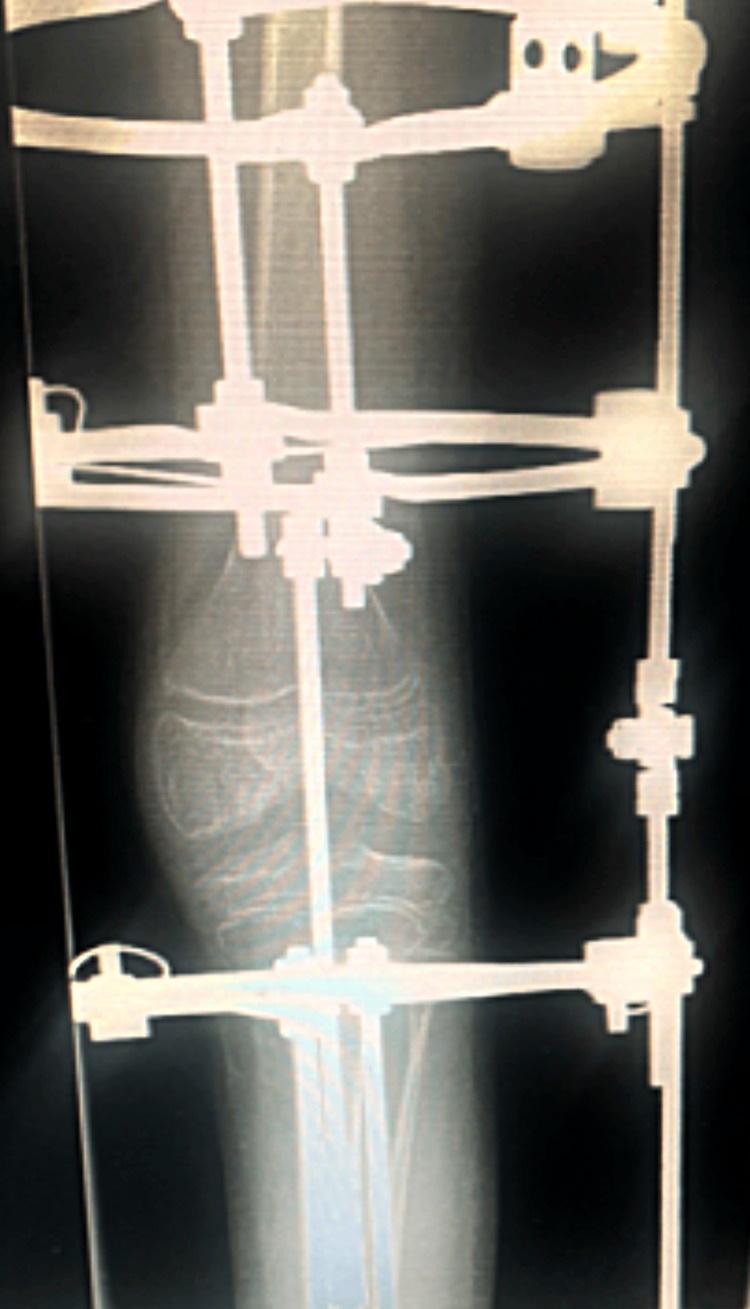
Postoperative anterior X-ray six weeks post-correction of the fixed flexion deformity of the left knee

**Figure 4:**
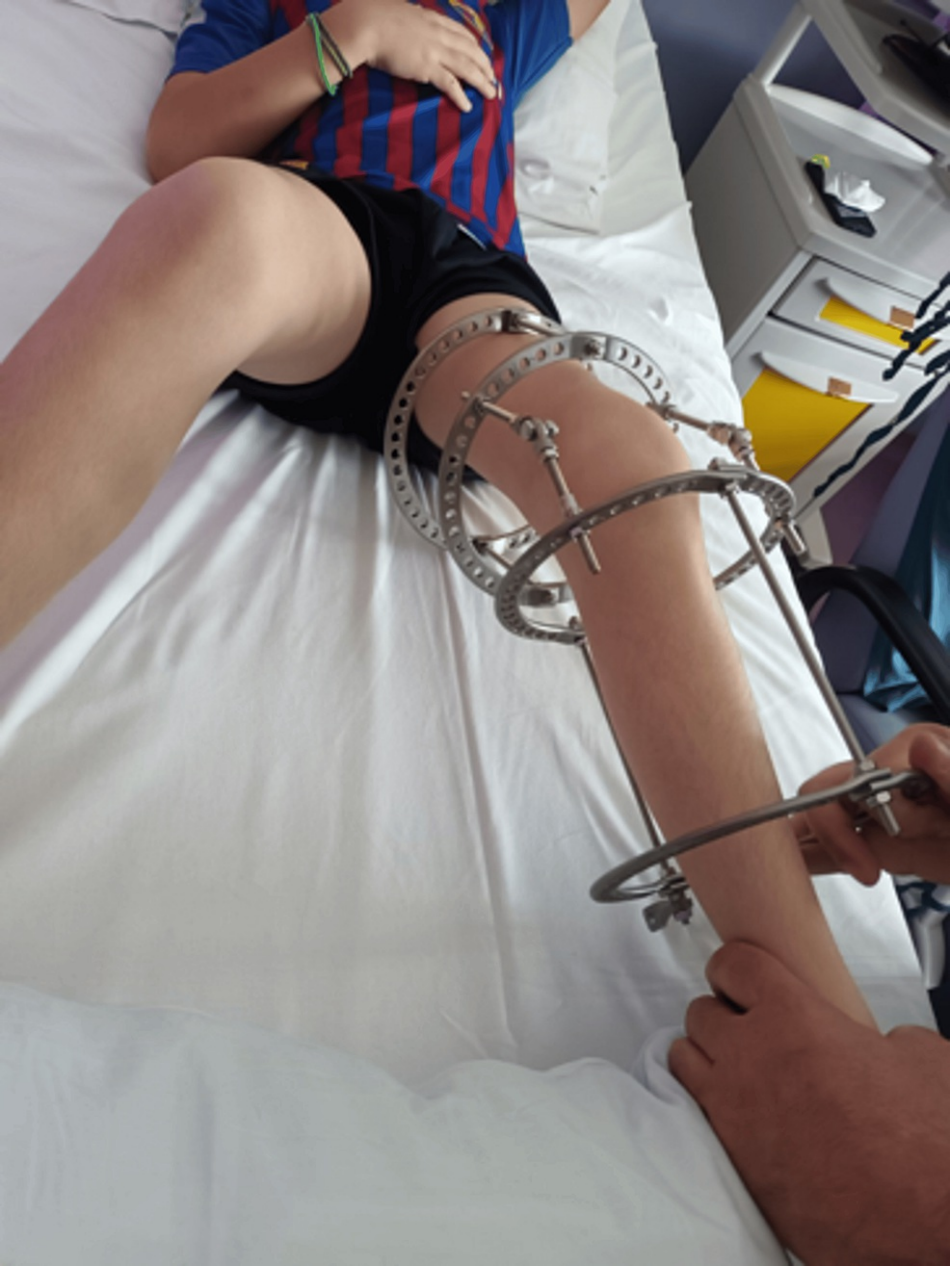
The Ilizarov apparatus in place, with the distraction rods controlling the progressive correction of the flexion contracture

A 12-year-old male with hemophilia A was monitored for knee effusions in his left knee; he also had a significant flexion contracture of 45°, with an inability to walk unaided as well as decreased participation in everyday activities. Hemophilia Early Detection Ultrasound (HEAD-US) showed severe synovitis and cartilage derangement while X-rays confirmed cartilage erosions as well as joint narrowing (Figure 1, Figure 2). The Ilizarov technique was chosen for surgical management of the worsening knee flexion contracture. The limbs were measured for the appropriate sizes, and the Ilizarov frames were constructed preoperatively. Application of the apparatus was performed under general anesthesia and fluoroscopic control for exact wire placement. Each frame consisted of two rings. The tibial fixation was similar to the femoral frame. The femoral and tibial components were joined by two distraction rods, using the knee joint as a hinge (Figure 3, Figure 4). Intraoperatively, no skin incisions are required, except for the placement of the pins and wires. The duration of the procedure was 55 minutes.

The boy had developed a high-responding FVIII inhibitor since 2012 when he was 18 months old, and he had never been treated by immune tolerance therapy. For the last two years, he was on prophylaxis with emicizumab subcutaneously once weekly while he was being treated with recombinant Factor VIIa (rFVIIa) in cases of acute bleeding. Concerning the perioperative plan, the patient was given one dose of rFVIIa shortly before the surgical operation and on a 2-3-4-6-8-hour schedule for a week after the orthopedic external fixator was placed around the patient's left knee while prophylaxis with emicizumab continued regularly. The rate of distraction was modified according to the patient's compliance, his feeling of pain, and physiotherapy management (Figure 3). When adequate extension of the left knee was achieved, the fixation device was removed and intensive physical rehabilitation was conducted to restore range of motion, muscle strength, proprioception, and gait pattern. An everyday evaluation was performed for the exclusion of signs of pin tract infection, checking for normal peripheral circulation and sensation, and measurement of the knee flexion angle and range of motion (Figure 4). The duration of distraction was six weeks. No pin tract infection was signed, and the patient had an uneventful postoperative discharge. However, after the Ilizarov device removal, the joint impairment returned, with decreased mobility. However, no bleeding, effusions, vascular injury, or bone fracture were assessed. Due to localized pain and functional impairment, the intra-articular administration of PRP was applied twice, on the first month after the circular frame removal and at a six-month follow-up. The VAS score of the patient sustained a beneficial effect despite the radiographic assessment. The fixed flexion deformity on the last follow-up of six months postoperatively was reduced from 45° to 5°. On the last follow-up, the patient was able to walk unaided and was delighted with the functional result.

## Discussion

The improvement in the field of prophylactic treatment of patients with hemophilia, during the last 20 years, has significantly decreased the incidence of severe hemophilic arthropathy in young boys who are treated prophylactically early after the diagnosis of the disease. Nevertheless, there are still some patients who suffer from many joint bleeds, especially those with high-titer FVIII inhibitors. These patients would develop severe arthropathy early in their adolescence, affecting mobility and quality of life, with the knees and ankles most frequently affected. The conservative treatment of fixed flexion deformities of the knee includes physiotherapy and orthotic devices [5]. However, in cases such as the one presented above, surgical intervention is indicated, as the fixed contracture of the knee had greatly deteriorated the quality of life of the patient. The proper correction to 5° degrees of flexion was achieved by the circular external fixator, and the preservation of the functional outcome was achieved by the intraarticular administration of PRP in association with his hemophilia prophylactic treatment by emicizumab. Also, the gradual daily distraction avoided the development of spontaneous hemorrhages and neurovascular injury. However, the most common complication with external fixators is the pin tract infections, which, in our case, did not happen [6].

Regarding PRP administration, it is defined as a volume of autologous plasma that contains a platelet concentration above the basal level in whole blood. PRP is potentially beneficial in tissue regeneration, with increasing applications in the musculoskeletal system mainly due to its anti-inflammatory role. In a study by Li et al., it was highlighted that intra‐articular PRP injection was successful in the treatment of hemophilic arthropathy of the knee joint, as long as it is administered for at least six months in hemophilia patients [7]. More specifically, PRP showed beneficial effects in pain reduction and joint range of motion, in concordance with our case [8]. In a recent case series, PRP treatment was found to provide sustained joint pain relief and improvement in the visual analog scale (VAS) and Western Ontario and McMaster University Osteoarthritis index (WOMAC) scores [9]. In the current literature, PRP administration is likely to provide a good option for the delay of further surgical interventions in the knee such as arthrotomy, especially in cases where the administration of hyaluronic acid (HA) was not efficient [10].

## Conclusions

We suggest that the circular external fixator is a safe, successful, and minimally invasive technique for the management of fixed flexion deformity of the hemophilic knee, in association with the intra-articular administration of platelet-rich plasma. However, further investigation is warranted to enlighten a standardized PRP protocol of administration and dosing regimens for hemophilic arthropathy, especially of the knee, in the pediatric population.
